# Characterization of the Recognition Specificity of BH2, a Monoclonal Antibody Prepared against the HLA-B27 Heavy Chain

**DOI:** 10.3390/ijms16048142

**Published:** 2015-04-13

**Authors:** Hui-Chun Yu, Kuang-Yung Huang, Ming-Chi Lu, Hsien-Lu Huang, Wei-Ting Liu, Wen-Chien Lee, Su-Qin Liu, Hsien-Bin Huang, Ning-Sheng Lai

**Affiliations:** 1 Department of Life Science and Institute of Molecular Biology, National Chung Cheng University, Chia-Yi 621, Taiwan; E-Mails: df928039@tzuchi.com.tw (H.-C.Y.); hky0919@yahoo.com.tw (K.-Y.H.); weiting0608@hotmail.com (W.-T.L.); 2 Section of Allergy, Immunology and Rheumatology, Department of Medicine, Buddhist DaLin Tzu-Chi Hospital, Chia-Yi 622, Taiwan; E-Mails: dm252940@tzuchi.com.tw (M.-C.L.); df897226@tzuchi.com.tw (S.-Q.L.); 3 School of Medicine, Tzu-Chi University, Hualien 970, Taiwan; 4 Department of Nutrition and Health Science, Fooyin University, Kaohsiung 831, Taiwan; E-Mail: estrus@mail2000.com.tw; 5 Department of Chemical Engineering, National Chung Cheng University, Chia-Yi 621, Taiwan; E-Mail: chmwcl@ccu.edu.tw

**Keywords:** HLA-B27, ankylosing spondylitis, monoclonal antibody, HLA-typing

## Abstract

BH2, a monoclonal antibody prepared against the denatured human leukocytic antigen-B27 heavy chain (HLA-B27 HC), can immunoprecipitate the misfolded HLA-B27 HC complexed with Bip in the endoplasmic reticulum and recognize the homodimerized HLA-B27 HC that is often observed on the cell membrane of patients suffered from ankylosing spondylitis (AS). However, the recognition specificity of BH2 toward the other molecules of HLA-B type and toward the different types of HLA molecules remained uncharacterized. In this study, we carried out the HLA-typing by using the Luminex Technology to characterize the recognition specificity of BH2 and analyzed the binding domain of HLA-B27 HC by BH2. Our results indicated that BH2 preferably binds to molecules of HLA-B and -C rather than HLA-A and the binding site is located within the α2 domain of HLA-B27 HC.

## 1. Introduction

The major histocompatibility complex (MHC) class I molecule is a heterodimeric protein that comprises a heavy chain (HC) and a light chain (β_2_-microglobulin, β_2_m). MHC class I molecules assemble with an antigenic peptide (approximate 8–12 amino acids in length) in the endoplasmic reticulum (ER) to form a heterotrimer and carry the bound peptide to the cell surface, where the peptide is displayed to cytotoxic T cells [1,2,3,4]. The HC (also called α chain), encoded by a human leukocytic antigen (HLA) gene, is polymorphic and is folded into three domains, α1, α2 and α3. Both α1 and α2 domains are associated together to form an antigen peptide-binding site, while the α3 domain containing a transmembrane segment is noncovalently associated with β2m at the extracellular surface. MHC class I molecules are classified by three major types, HLA-A, HLA-B and HLA-C. Their genes are clustered on human chromosome 6 and their protein sequences share a high identity [5,6,7,8].

Ankylosing spondylitis (AS) is a chronic systemic inflammatory disease [9,10,11,12,13]. AS is highly linked with the expression of HLA-B27 [14,15]. Up to 95% of AS patients are HLA-B27-positive. Several lines of evidence have demonstrated that the HLA-B27 heavy chain (HLA-B27 HC) has an intrinsic propensity to fold slowly in the ER before it is assembled with β_2_m and a peptide, in turn leading to the formation of disulfide-linked heavy-chain homodimers, (B27-HC)_2_ [16,17,18]. (B27-HC)_2_ is permitted to leave the ER, trafficked to the cell surface [16,17,18] and displayed to the natural killer-cell Ig-like receptor (KIR3DL2) as well as to T-helper 17 cells (Th17), in turn stimulating their activation and leading to one of the major pathogenic potentials in AS [19,20,21]. Thus, the levels of (B27-HC)_2_ can be an index of the misfolded form of HLA-B27 HC. In our previous study, we have prepared an anti-HLA-B27 HC monoclonal antibody, BH2 [22]. The misfolded HLA-B27 can form a complex with Bip in the ER [10,23]. BH2 can immunoprecipitate the misfolded HLA-B27/Bip complex and recognize the disulfide-linked (B27-HC)_2_ [22]. However, the recognition specificity toward different HLA class I alleles by BH2 needed to be characterized. In this study, we characterize the binding specificity of BH2.

## 2. Results

### 2.1. Recognition Specificity of BH2

**Figure 1:**
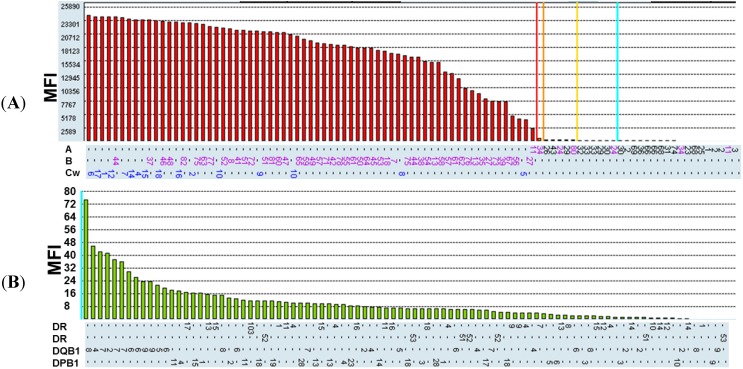
Characterization of binding specificity for BH2 by human leukocytic antigen (HLA)-typing. (**A**) Analysis of BH2 binding to major histocompatibility complex (MHC) class I molecules by using the Luminex Technology. MFI represents mean fluorescence intensity. Cut-off values of mean fluorescence intensity (MFI) for positive binding were set at 637; (**B**) Analysis of BH2 binding to MHC class II molecules by using the Luminex Technology. Cut-off values of MFI for positive binding were set at 500.

There are three major MHC class I gene clusters located on chromosome 6 of human, encoding HLA-A, HLA-B and HLA-C heavy chain molecules. DNA or protein sequences among these three different types of HLA molecules share a high identity. BH2 can recognize the misfolded HLA-B27 HC. However, the recognition specificity of BH2 toward the other HLA-B molecules or toward different types of HLA molecules remained unknown. Thus, we carried out HLA typing for BH2 by using the Luminex Technology to analyze its recognition specificity. Each individual microbead was conjugated with the unique HLA molecule antigen. The mixtures of microbeads consisting of HLA molecules were incubated with BH2, reacted with R-phycoerythrin-conjugated secondary antibodies and then analyzed by flow cytometry. Value of mean fluorescence intensity (MFI) for different types of HLA antigens bound by BH2 is shown in Figure 1A. Cut-off values of MFI for HLA molecules were set at 637. MFI value more than 637 was considered as a positive binding of HLA molecule by BH2. The results show that BH2 binds to HLA-B and HLA-C molecules. However, BH2 only binds to HLA-A11, but not to other HLA-A antigens in our assay. HLA-B, -C, and -A molecules recognized by BH2 are summarized in Table 1. In addition, BH2 binding to MHC class II molecules were also analyzed by the same technique using microbeads coated with MHC class II antigens. Value of MFI for each type of MHC class II molecules are shown in Figure 1B. Cut-off values of MFI for this assay were set at 500. MFI value of more than 500 was suggested to be a positive binding between BH2 and MHC class II molecule. All of MFI values are less than 500, suggesting that BH2 cannot recognize MHC class II molecules.

**Table 1:** MHC class I molecules, not class II molecules, bind to BH2. The results were summarized from Figure 1A,B.

Class I (Positive)	Class II (Positive)
Cw10, B27, B57, B51, B61, B13, B44	****
B75, B55, Cw4, Cw12, B49, B38, Cw1	****
B76, B54, Cw16, B8, B48, B35, Cw15	****
B42, Cw7, Cw9, B41, B7, B62, Cw6	****
B82, B37, Cw17, B60, B56, B65, B45, B81	**Negative**
B59, B52, B39, Cw2, Cw14, B78, B46	****
Cw5, Cw8, B72, B58, B63, B77, B73	****
B71, B64, B53, B67, B47, B18, Cw18	****
B50, A11	****

### 2.2. BH2 Binds to the α2 Domain of HLA-B2704 HC

**Figure 2:**
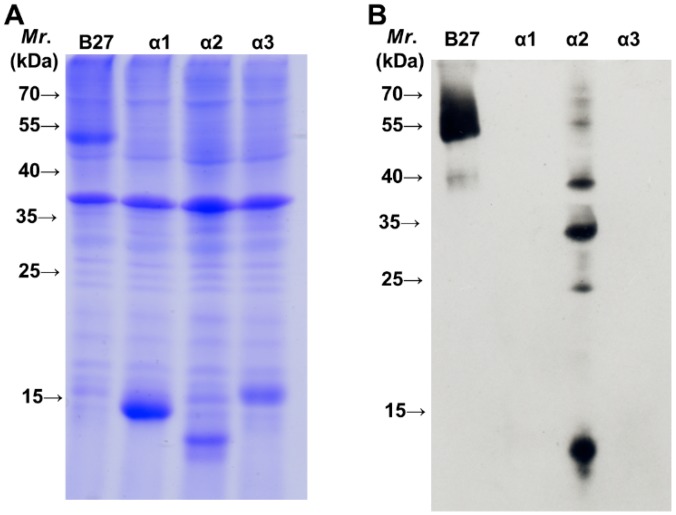
Analysis of HLA-B27 heavy chain domain recognized by BH2. (**A**) SDS-PAGE analysis of HLA-B27 domains overexpressed in *E. coli* (BL21 DE3); (**B**) Domain of HLA-B27 heavy chain recognized by BH2 was analyzed by western blotting. An aliquot (20 µg) of each crude protein extracted from *E. coli* bacteria that have overexpressed the indicated domain of HLA-B27 HC was separated by SDS-PAGE (15%) and analyzed by western blotting using BH2 monoclonal antibody.

**Figure 3:**
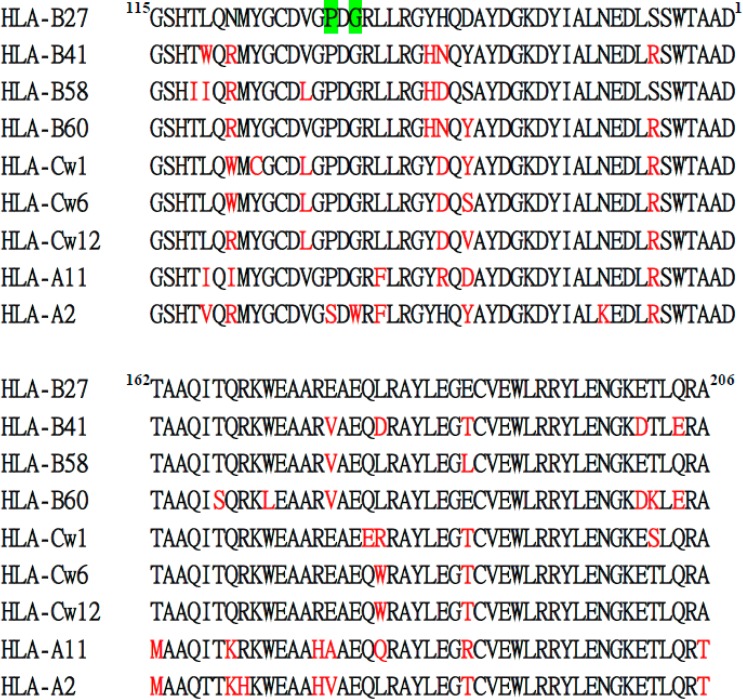
Amino acid sequence alignment of HLA-B27 HC α2 domain with that of the indicated HLA-B, -C, and -A molecules. BH2 binds to HLA-B27, -B41, -B58, -B60, -Cw1, -Cw6, -Cw12 and -A11, but not to HLA-A2 in HLA-typing assay. The non-consensus residues compared with HLA-B27 are marked with red. Double replacements of Pro-129 and Gly-131 of HLA-B27 HC with Ser and Trp, respectively, are marked as green.

**Figure 4:**
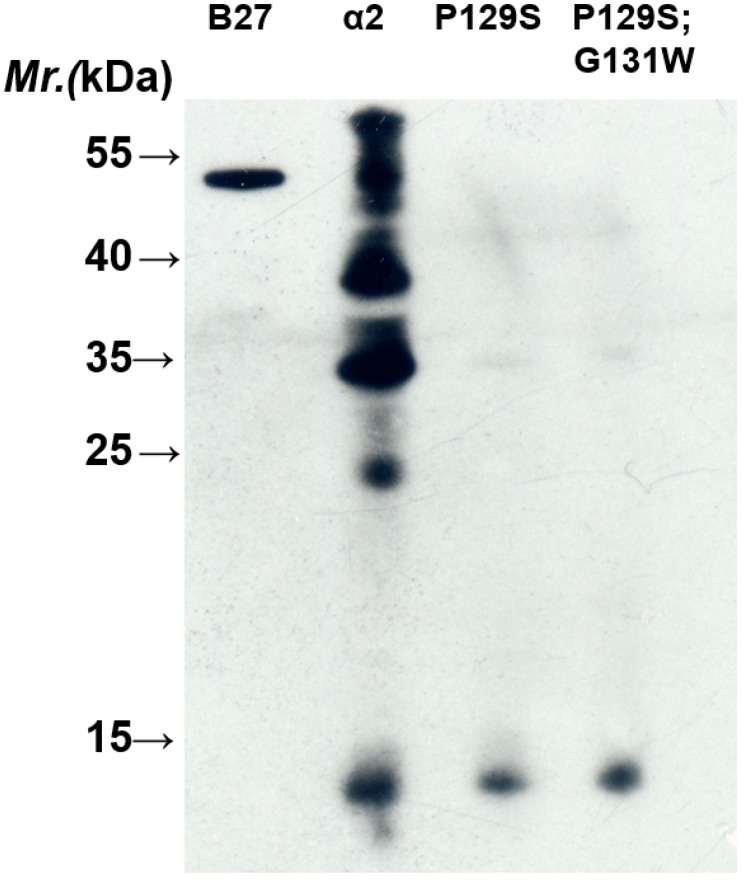
Double replacements of Pro-129 and Gly-131 with Ser and Trp, respectively, on the α2 domain do not affect the BH2-binding. An aliquot (20 µg) of each crude protein extracted from *E. coli* bacteria that have overexpressed the indicated mutant α2 domain of HLA-B27 HC was separated by SDS-PAGE (15%) and analyzed by western blotting using BH2 monoclonal antibody.

The heavy chain of MHC class I molecules from *N*- to *C*-terminus consists of three domains, α1, α2 and α3, each about 90 residues in length. Both α1 and α2 domains fold into a module to form an antigenic peptide binding site. The α3 domain connected to the transmembrane domain via a short flexible linker is a site for the non-covalent interaction with β_2_m at the extracellular surface. We also analyzed which domain of HLA-B27 HC is recognized by BH2. Each domain of HLA-B2704 HC was cloned and over-expressed in *E. coli* (BL21 DE3). Figure 2A indicates the expression of each domain induced by isopropyl β-d-1-thiogalactopyranoside (IPTG). Western blotting analysis demonstrated that BH2 binds to the α2 domain (Figure 2B), but not to α1 and α3 domains. The recombinant α2 domain of HLA-B27 HC showing multiple bands on SDS-PAGE (Figure 2B) may arise from formation of the inclusion body or from the aggregated forms. We randomly picked up some α2 domain of HLA class I alleles for sequence alignment analysis to figure out the potential binding epitope by BH2 (Figure 3). BH2 recognizes HLA-B27, -B41, -B15, -Cw1, -Cw6, -Cw12 and -A11, but not HLA-A2. Based on the sequence alignment of α2 domains, we hypothesized that Pro-129 and Gly-131 of α2 domain could play a critical role in BH2 binding. However, after replacing Pro-129 and Gly-131 of α2 domain in HLA-B27 by Ser and Trp, respectively, using site-directed mutagenesis. The mutant α2 domain of HLA-B27 remained bound to BH2, seen by western blotting assay (Figure 4). HC10 is one of the monoclonal antibodies that were prepared against a mixture of denatured HLA-B7 and -B40 heavy chains [23]. HC10 can immunoprecipitate the misfolded HLA-B27/Bip complex [10,24] and recognize the homodimeric HLA-B27, (B27-HC)_2_ [24]. Although it is still unknown which of the α domains of HLA-B27 HC is recognized by HC10, HC10 prefers to bind to HLA-B and HLA-C types than to HLA-A type [24]. Both BH2 and HC10 can recognize the misfolded HLA-B27 HC, but their binding specificity toward HLA-A loci is subtly different. HC10 recognizes HLA-A3 and -A33 [23]. However, in our HLA-typing, BH2 fails to recognize HLA-A3 and -A33. Up to now, only HC10 and BH2 have been proved to recognize the misfolded HLA-B27 HC and (B27-HC)_2_.

## 3. Experimental Section

### 3.1. Materials

Dithiothreitol (DTT), Tris, Luria-Bertani (LB) broth, kanamycin, isopropyl β-d-1-thiogalactopyranoside (IPTG), sodium dodecyl sulfate (SDS), TEMED, ammonium persulfate, acrylamide, glycine and Tris Base were obtained from Sigma-Aldrich (St. Louis, MO, USA).

### 3.2. HLA-Typing

HLA recognition specificity of BH2 was characterized following the methods as described by the manufacturer (Luminex Corporation, Austin, TX, USA) [25]. Briefly, 10 µL of LABScreen Mixed kit (One Lambda, Canoga Park, CA, USA) containing microbeads coated with purified Class I or Class II HLA antigens were incubated with 30 µL of BH2 monoclonal antibody in the dark at room temperature for 30 min. All components were washed with the buffer to remove the unbound BH2. The antibody bound to the antigen coated on the microbeads was reacted with *R*-phycoerythrin-conjugated goat anti-mouse IgG. The fluorescent emission on the microbeads was detected by the LABScan 100 flow analyzer (Canoga Park, CA, USA) and analyzed by One Lambda software (Canoga Park, CA, USA). Cut-off values were established according to the manufacturer’s instructions.

### 3.3. Analysis of BH2-Binding Domain

HLA-B27 HC consists of three domains (α1, α2 and α3). The full-length of human HLA-B27 HC cDNA was served as a template and cDNA of each domain was cloned by PCR using the following primers:

Alpha 1 primers: 5'-GGCGGCTCCCACTCCATGAGG-3' and 5'-GGCCTCGCTCTGGTTGATG-3'
Alpha 2 primers: 5'-GGGTCTCACACCCTCCAGAA-3' and 5'-CGCGCGCTGCAGCGTCTC-3'
Alpha 3 primers: 5'-GACCCCCCAAAGACACACG-3' and 5'-CCATCTCAGGGTGAGGGG-3'

The resulting product was cleaved by BamHI/EcoRI and subcloned into the pET28a plasmid. *E. coli* (BL21 DE3) cells transformed with the recombinant vector encoding α1, α2 or α3 domain of B27 HC were grown in 5 mL of LB broth at 37 °C for 3 h. Then, protein expression was induced by IPTG (final concentration of 1 mM) for 3 h. Bacteria (1 mL) were subjected to centrifugation at 12,000× *g* for 3 min and the supernatant was discarded. The pelleted cells were re-suspended by 100 µL of 1% SDS and ruptured by ultrasonication. An aliquot (20 µL) of extracted proteins was separated by SDS-PAGE (15%) and analyzed by western blotting using BH2 monoclonal antibody.

### 3.4. Site-Directed Mutagenesis

Site-directed mutagenesis was carried out by using the QuikChange Site-directed Mutagenesis Kit (Stratagene, La Jolla, CA, USA) with the primers (α2 domain P129S: 5'-GGCTGCGACGTGGGGTCGGACGGGCGCCTCCTCCGC-3'; α2 domain P129S; G131W: 5'-GGCTGCGACGTGGGGTCGGACTGGCGCCTCCTCCGCGGG-3') following the methods described by the manufacturer. The plasmids, pET28a-α2 domain and pET28a-α2 domain P129S, were used as the templates for site-directed mutagenesis to produce the plasmids, pET28a-α2 domain P129S and pET28a-α2 domain P129S; G131W, respectively.

## 4. Conclusions

We have demonstrated that BH2 prefers to bind to HLA-B and HLA-C rather than to HLA-A types in HLA typing assay (Figure 1A). BH2 binds to HLA-A11, but show a more weak binding affinity. No other HLA-A molecules were observed to interact with BH2 in our assays (Figure 1A). BH2 binds to the α2 domain of HLA-B27. However, the recognition site in α2 domain of HLA-B27 HC by BH2 needs to be characterized.
